# Recombinant HBHA Boosting Effect on BCG-Induced Immunity against *Mycobacterium tuberculosis* Infection

**DOI:** 10.1155/2011/730702

**Published:** 2011-05-23

**Authors:** G. G. Guerrero, C. Locht

**Affiliations:** ^1^INSERM U1019, Lille, France; ^2^CNRS UMR8204, Lille, France; ^3^Institut Pasteur de Lille, Lille, France; ^4^Université Lille Nord de France, Lille, 59000, France; ^5^Facultad de Ciencias Químicas, UJED, Gómez Palacio, DGO. 35060, Mexico

## Abstract

Heterologous prime-boost regimens are effective strategies to promote long-term memory and strong cellular Th1 responses to *Mycobacterium tuberculosis*, when BCG is used in the priming step. Subcutaneous or intranasal boosting of BCG-vaccinated newborn mice with native heparin-binding haemagglutinin (nHBHA) significantly enhances protection against *M. tuberculosis.* However, nHBHA is characterized by a complex methylation pattern in its C-terminal domain, which is important for protective immunogenicity in primary vaccination. In this study we addressed the question whether boosting with recombinant, non-methylated HBHA (rHBHA) produced in *Escherichia coli* may enhance protection of BCG-primed newborn mice. We found that while subcutaneous rHBHA boosting enhanced protection of BCG-primed mice against intranasal *M. tuberculosis* infection both in spleen and lungs, enhanced protection against aerosol infection was only seen in the spleen (0.72 logs; *P* < 0.05) but not in the lungs. Thus, in BCG-primed mice the methylation of the C-terminal domain of HBHA is dispensable for the induction of enhanced protection in the lungs against intranasal but not aerosol infection, whereas it enhances protection in the spleen in both challenge models. This report thus provides evidence that rHBHA may be considered as a booster vaccine against disseminated tuberculosis.

## 1. Introduction

Tuberculosis (TB) remains one of the main causes of mortality in the world [1, 2]. *Mycobacterium bovis* Bacille Calmette-Guérin (BCG) [3], a live, attenuated mycobacterial strain is still the only vaccine available against TB. While it has clear beneficial effects against pulmonary and disseminated TB in children for a limited number of years, it provides insufficient protection against pulmonary tuberculosis in adults, with highly variable protective efficacy [1, 4]. Therefore, more effective vaccines to increase or boost BCG-induced immune protection against TB are urgently needed. Recent advances have shown that non-living vaccines based on secreted proteins and prime-boost strategies [5, 6] can effectively protect against subsequent *Mycobacterium tuberculosis* infection in animal models and increase immune responses in humans [7–11].

The most desirable immunological consequence of the vaccination with BCG is the priming of a Th1-oriented CD4^+^ T cell response that maximizes, through the release of IFN-*γ*, the antimicrobial properties of macrophages [12–14]. In this sense, it has been demonstrated that the mycobacterial adhesin heparin-binding haemagglutinin (HBHA) [15, 16] is a promising antigen against TB [17, 18]. HBHA is a methylated protein, and the methylation pattern in its C-terminal domain is important for the production of HBHA-specific IFN-*γ* by peripheral blood mononuclear cells (PBMCs) from *M. tuberculosis*-infected healthy individuals [17]. In mouse models, methylated HBHA, but not recombinant, non-methylated HBHA (rHBHA), co-administered with the adjuvant dimethyl dioctadecylammonium bromide monophosphoryl lipid A (DDA+MPL) induced protection against intravenous (i.v.) or aerosol challenge with *M. tuberculosis* at levels similar to those induced by BCG [17–19], indicating the importance of the methylated C-terminal domain of HBHA for protection. However, the N-terminal domain is predicted to contain helix-coiled coil motifs, which are usually rich in T and B cell epitopes [20, 21]. Consistent with this, we have previously reported that rHBHA without adjuvant, administered to adult BALB/c mice by the intranasal (i.n.) or subcutaneous (s.c.) route, induces significant humoral and T cell immune responses [22], although protective immunity was not observed. On the other hand, we have also shown that native HBHA administered by the s.c. or i.n. route without adjuvant significantly enhances BCG-primed immunoprotection against *M. tuberculosis* infection [23]. In this study, we investigated whether; in a similar heterologous prime-boost regimen [23], rHBHA boosting confers enhanced protection against aerosol or i.n. *M. tuberculosis* infection in BCG-primed mice. We found that the methylation of the C-terminal domain of HBHA can be dispensable for the boosting effect of BCG-induced protective immunity in the lungs against i.n., but not aerosol *M. tuberculosis* infection, whereas it enhances protection in the spleen in both infection models. We therefore conclude that rHBHA may be considered as a booster vaccine to improve the efficacy of BCG for the prevention of disseminated TB to non-pulmonary locations. 

## 2. Material and Methods

### 2.1. Animals

Pathogen-free newborn (three to four days old) BALB/c mice were obtained from Harlan, Co, France and were maintained in a specific pathogen-free environment at the Institut Pasteur of Lille throughout the entire experiment. All animal experiments were performed according to the protocols approved by the Institutional Animal Care and Use Committee and mainly under barrier conditions in a level II and III biosafety animal facility.

### 2.2. Microorganisms

The BCG Pasteur strain (isolate 1173P2, World Health Organization, Stockholm, Sweden) was grown in dispersed cultures in Sauton medium for 14 days as previously described [15]. The BCG suspension was then stored at –80°C until further use. *M. tuberculosis* H37Rv used to challenge mice was grown at 37°C in Middlebrook 7H9 medium (DIFCO), supplemented with albumin-dextrose-catalase enrichment and Tween 80, collected at the end of the stationary phase and stored in Middlebrook 7H9 medium supplemented with 20% (v/v) glycerol at –80°C until they were used.

### 2.3. Immunizations

Recombinant HBHA was purified from *Escherichia coli* BL21(DE3)(pET-HBHA) [16] by using heparin-sepharose chromatography, followed by reverse phase high-pressure liquid chromatography, as previously described [15, 24]. Groups of 3-4-day-old neonate BALB/c mice were immunized s.c. [23] with 5 × 10^5^ colony-forming units (CFU) BCG in 50 *μ*L sterile phosphate-buffered saline (PBS) or mock-immunized with 50 *μ*L of sterile PBS. At intervals of one month, for a total of three months, each group of mice was boosted s.c. with 5 *μ*g rHBHA, dissolved in 150 *μ*L of PBS or as control received only PBS [23]. Mice were rested one month before *M. tuberculosis *challenge infection.

### 2.4. *M. tuberculosis* Challenge

For the i.n. challenge experiments, mice were first anesthetized with an intra-peritoneal (i.p.) injection of sodium pentobarbital (1/10 of the weight mouse) and then infected i.n. with 5 × 10^5^ CFUs of *M. tuberculosis* in 20 *μ*L of PBS. Alternatively, mice were aerosol-challenged by using a homemade nebulizer. 3 mL of a suspension containing 2 × 10^7^ CFUs was aerosolized to deliver an inhaled dose of 100–150 CFUs per mouse. Numbers of live bacteria in organs were measured eight weeks after infection by plating serial dilutions of whole organ homogenates on Middlebrook 7H11 solid medium supplemented with 2 mg/mL of THF. Petri dishes were kept in sealed plastic bags at 37°C for 3-4 weeks. Colonies were counted and total CFU calculated.

### 2.5. Cytokine Analyses

For cytokine measurements single cell suspensions of spleen or lung cells were prepared as described elsewhere [22]. Briefly, spleen tissues were smashed in a Falcon sieve using the top end of a 1 mL syringe piston. The excised lung tissue was minced and incubated for 1 h at 37°C in 500 *μ*L of PBS containing 2% fetal bovine serum (FBS) (Buffer I), 125 U/mL collagenase I (Sigma-Aldrich), and 60 U/mL DNase I (Sigma-Aldrich). Single cell suspensions were prepared by passing the tissue through a 30-mm stainless steel mesh, washed, resuspended in Buffer I, applied carefully onto a gradient of percoll (Sigma-Aldrich), and centrifuged at 2200 rpm for 30 min at 20°C. Live cells at the interface were collected and used for *in vitro* culture. 1 × 10^6^ viable cells were cultured in medium consisting of RPMI 1640 (GIBCO BRL) supplemented with 10% heat-inactivated FBS, 2 mM L-glutamine, 10 mM HEPES, 100 U of penicillin G per mL, 100 *μ*g of streptomycin per mL, and 0.05 mM 2-mercaptoethanol. Cells were incubated at 37°C in an atmosphere of 5% CO_2_ for 72 h in 96-well ELISA plates in the presence or absence of ConA (2.5 *μ*g/mL) or rHBHA (2.5 *μ*g/mL). The culture supernatants were then collected and stored at −20°C until analysis. The amounts of IL-6, IFN-*γ*, IL-12p70, and IL-10 in the supernatants were measured by using a specific sandwich ELISA (OptEIA; BD Biosciences-Pharmingen) according to the manufacturer's instructions. Assay sensitivities were 2.5 pg/mL for IFN-*γ* and 3 pg/mL for IL-6, IL12p70, and IL10. The data are expressed as the mean ± SEM for each mouse group.

### 2.6. Statistical Analysis

Differences between the different groups (three) were assessed by one-way analysis of variance (ANOVA) and by its nonparametric equivalent Kruskal-Wallis test of the log_10_ CFU followed by Tukey and/or Dunn's post-test, respectively. A value of *P* < 0.05 was considered statistically significant. Analysis was performed using Prism 5.0 software (Graph Pad Software, Inc., Sand Diego, Calif, USA).

## 3. Results

### 3.1. The Methylation Pattern in the C-Terminal Domain of HBHA Is Dispensable to Boost Protection of BCG-Vaccinated Newborn Mice against *M. tuberculosis * i.n. Challenge

The natural methylation pattern in the C-terminal domain of HBHA is important for T cell antigenicity and protective immunity [17]. No protective immunity was induced when rHBHA was co-administered with the adjuvant DDA+MPL to adult BALB/c or C57BL/6 mice challenged i.v. or through the aerosol route with *M. tuberculosis* [17, 18]. However, it has been shown that heterologous prime-boost regimens are efficient to boost BCG-induced immunity against *M. tuberculosis* [5, 9]. We have recently reported that boosting (i.n. or s.c.) with nHBHA in the absence of adjuvant was an efficient way to significantly enhance BCG-induced immunoprotection against *M. tuberculosis* infection [23]. Taking into account that rHBHA is easily purified from recombinant *E. coli*, we investigated whether this form of HBHA may also boost BCG-induced protective immunity. Therefore, newborn mice were vaccinated with BCG and then boosted with rHBHA by the s.c. route. One month after the last immunization, the mice were infected with *M. tuberculosis* and sacrificed 8 weeks after challenge to count the CFUs in spleen and lungs, as a measure of protection. BCG-primed/rHBHA-boosted mice had a significantly reduced bacterial load in the spleen (by 1.32 logs, *P* < 0.05) in comparison with PBS control mice (3.72 ± 0.12 versus 5.04 ± 0.29) (Table 1), which was significantly lower than in BCG-vaccinated, non-boosted animals (by 0.89 logs, *P* < 0.05; 3.72 ± 0.12 versus 4.15 ± 0.55) (Table 1). Furthermore, in the lungs BCG-vaccinated/rHBHA-boosted mice had also significantly reduced bacterial loads compared to the PBS controls (by 2.26 logs, *P* < 0.05; 3.98 ± 0.11 versus 6.24 ± 0.25) and compared to BCG-vaccinated non-boosted mice (by 1.26 logs, *P* < 0.05; 3.98 ± 0.11 versus 5.24 ± 0.40) (Table 1). These data indicate that the methylation pattern in the C-terminal domain of HBHA is dispensable for the booster effect of BCG-induced protective immunity after i.n. *M. tuberculosis* infection.

### 3.2. The Methylation Pattern in the C-Terminal Domain of HBHA Is Important to Boost Lung Protection of BCG-Vaccinated Newborn Mice against *M. tuberculosis* Aerosol Challenge

Next, we evaluated the rHBHA boosting effect of the BCG-induced protective immunity against *M. tuberculosis *aerosol infection. BCG-vaccinated newborn mice boosted with rHBHA were challenged by aerosol with a low dose of *M. tuberculosis* (*∼*100–150 CFUs). Eight weeks post-challenge, BCG-vaccinated/rHBHA-boosted mice showed a significant reduction of bacteria in the spleen (by 1.35 logs, *P* < 0.05) in comparison with PBS control mice (3.15 ± 0.71 versus 4.50 ± 0.46) (Table 1), as well as in comparison with BCG-vaccinated, non-boosted mice (by 0.72 logs, *P* < 0.05; 3.15 ± 0.71 versus 3.87 ± 0.34) (Table 1). In contrast, in the lungs, both BCG-vaccinated non-boosted and BCG-vaccinated/rHBHA-boosted mice showed reduced bacterial load at a similar magnitude in comparison to the PBS control mice (by 0.59 logs) (Table 1). Thus, in contrast to the i.n. *M. tuberculosis *infection, upon aerosol challenge rHBHA boosting did not enhance BCG-induced protective immunity in the lung, while it boosted protection in the spleen. These data indicate therefore that natural methylation present in the C-terminal of nHBHA and absent in rHBHA is important to enhance BCG-induced protective immunity in the lungs, but not in the spleen against *M. tuberculosis *aerosol-infection.

### 3.3. rHBHA Boosting of BCG-Induced Protective Immunity against i.n. Challenge Correlates with High IL-12 Production

We have previously reported that in the absence of DDA+MPL, nHBHA boosting of the BCG-induced protective immunity against i.n. or aerosol *M. tuberculosis* infection correlates with high IL-12 and TGF-*β* production [23], two cytokines that are important for the maintenance of the memory T cell population [25–27], especially when boosting occurs late after priming. Therefore, we determined whether the rHBHA-mediated booster effect of the BCG-induced protective immunity after *M. tuberculosis *infection (either i.n./or aerosol) also correlates with these two cytokines induced after challenge in a long-term prime boost protocol and *in vitro* recall with rHBHA. 

**Figure 1:**
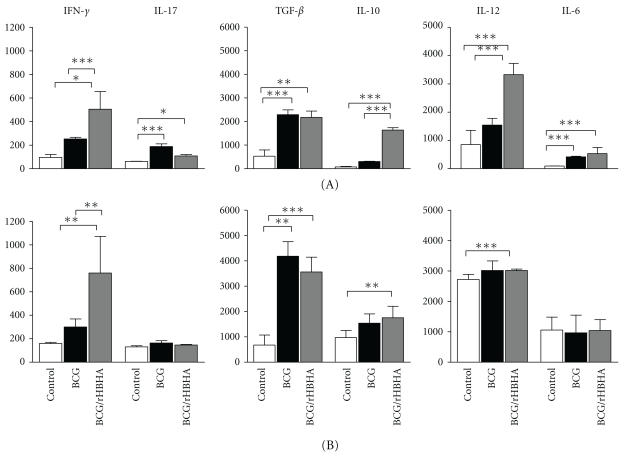
Spleen cell cytokine profile of BCG-vaccinated mice with or without rHBHA boost after *M. tuberculosis* infection. Eight weeks after i.n. (A) or aerosol (B) challenge spleen cells from control mice (white bars), BCG-vaccinated mice (black bars), or BCG-vaccinated/rHBHA-boosted mice (gray bars) were cultured in the presence of medium only, ConA (2.5 *μ*g/mL), or rHBHA (5 *μ*g/mL). Levels of cytokines were measured after 72 h culture in the supernatants by using the OptEIA kit (BD Biosciences). Values are expressed in pg/mL and represent media ± SEM of samples tested in duplicates from each group of mice. The data was considered statistically significant when *P* < 0.05 (*), *P* < 0.001 (**), and *P* < 0.0001 (***).

Splenocytes from i.n. infected mice after *in vitro* recall with rHBHA produced significant amounts of IL12p70 (3325 ± 403 pg/mL) (Figure 1A, gray bar), in comparison with PBS-immunized mice (850 ± 508 pg/mL) (Figure 1A, white bar) and BCG-vaccinated non-boosted mice (1540 ± 239 pg/mL) (Figure 1A, black bar). In contrast, the spleen cell TGF-*β* production in BCG-vaccinated/rHBHA-boosted mice after infection was similar (2174 ± 270 pg/mL) to that of spleen cells from the BCG-vaccinated i.n. infected mice without boost (2291 ± 203 pg/mL) (Figure 1A). BCG-vaccinated/rHBHA-boosted mice also produced slightly higher amounts of IFN-*γ* (505 ± 150 pg/mL, *P* < 0.05) (Figure 1A) after infection, in comparison with those induced by the BCG-vaccinated non-boosted mice (252 ± 14 pg/mL) (Figure 1A) and PBS control mice (236 ± 32) (Figure 1A). We also determined regulatory (IL-10) and Th17 type cytokines (IL-6, IL-17) and found that splenocytes from BCG-vaccinated/rHBHA-boosted i.n. infected mice induced a significant IL-10 production (1640 ± 100 pg/mL) in comparison with infected PBS control mice (76 ± 20 pg/mL) and infected BCG-vaccinated mice without boost (304 ± 14 pg/mL) (Figure 1A). Interestingly, the IL-6 spleen cell production from BCG-vaccinated/rHBHA-boosted i.n. infected mice (427 ± 89 pg/mL) was similar to that of the non-boosted group (419 ± 20 pg/mL), but significantly higher than that of the PBS control mice (96 ± 4 pg/mL) (Figure 1A). Spleen cell IL-17 production significantly changed between the three groups after i.n. infection (Figure 1A).

After aerosol infection, splenocytes from BCG-vaccinated/rHBHA-boosted mice produced similar amounts of IL-12p70 (3020 ± 42 pg/mL) as those from BCG-vaccinated non-boosted mice (3020 ± 313 pg/mL) (Figure 1B), but higher than those from the PBS control mice (2720 ± 170 pg/mL) (Figure 1B). A similar pattern was observed for the spleen cell TGF-*β* production (BCG: 1540 ± 367 pg/mL; BCG/rHBHA: 1755 ± 450 pg/mL; controls: 970 ± 403 pg/mL). Interestingly, after aerosol infection, spleen cells from BCG-vaccinated/rHBHA-boosted mice produced also a modest but significant amount of IFN-*γ* (760 ± 312 pg/mL) (Figure 1B, gray bar), higher than spleen cells from BCG-vaccinated mice without boost (300 ± 68 pg/mL) (Figure 1B, black bar) and PBS control mice (159 ± 10 pg/mL) (Figure 1B, blank bars) (*P* < 0.05). The spleen cell production of IL-10, IL-17, and IL-6 was not significantly different between the different groups of aerosol-infected mice (Figure 1B).

Thus it appears that for some cytokines the booster effect of rHBHA upon BCG priming depends on the route of infection (i.n. for IL-12, IL-10; aerosol and i.n. for IFN-*γ*).

### 3.4. Th1-Type Cytokine Induction in the Lungs from BCG-Vaccinated/rHBHA-Boosted Mice after Infection

To determine whether the enhanced lung protective effect of BCG-vaccinated/rHBHA-boosted mice against i.n. infection compared to BCG-vaccinated, non-boosted mice also correlates with high IL-12 and/or TGF-*β* production, eight weeks after challenge (either i.n. or aerosol), the cytokine production was determined in the supernatant of the lung cell cultures after *in vitro* recall with rHBHA.

**Figure 2:**
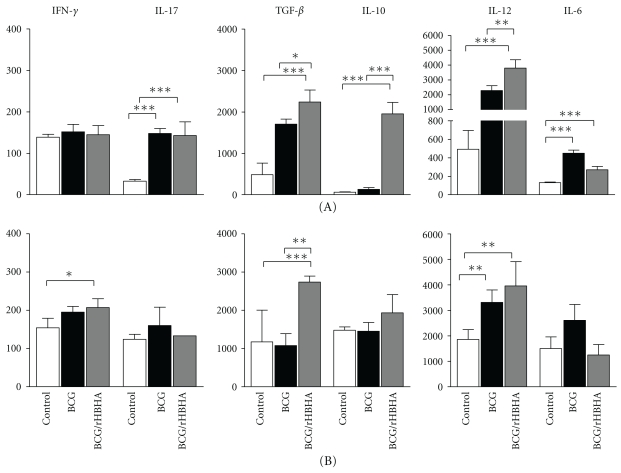
Lung cell cytokine profile of BCG-vaccinated mice with or without rHBHA boost after *M. tuberculosis* infection. Eight weeks after i.n. (A) or aerosol (B) challenge lung cells from control mice (white bars), BCG-vaccinated mice (black bars), or BCG-vaccinated/rHBHA-boosted mice (gray bars) were cultured in the presence of medium only, ConA (2.5 *μ*g/mL), or rHBHA (5 *μ*g/mL). Levels of cytokines were measured after 72 h culture in the supernatants by using the OptEIA kit (BD Biosciences). Values are expressed in pg/mL and represent media ± SEM of samples tested in duplicates from each group of mice. Statistically significant data: *P* < 0.05 (*), *P* < 0.001 (**), and *P* < 0.0001 (***).

Lung cell IL-12 production of BCG-vaccinated/rHBHA-boosted mice infected i.n. was significantly higher (3800 ± 560 pg/mL) (Figure 2A) than that of non-boosted mice (2280 ± 335 pg/mL) or PBS control mice (433 ± 222 pg/mL) (Figure 2A). Interestingly, the lung cells from BCG-vaccinated/rHBHA-boosted mice also produced higher amounts of TGF-*β* (2240 ± 290 pg/mL) after i.n. infection, in comparison with non-boosted mice (1707 ± 122 pg/mL) and the controls (489 ± 277). The lung cell IL-10 production was significantly higher in BCG-vaccinated/rHBHA-boosted mice (1955 ± 275 pg/mL) than in non-boosted (252 ± 42 pg/mL) and in control mice (68 ± 7 pg/mL) after i.n. infection (Figure 2A). Very low levels of IFN-*γ* were produced by lung cells in all three groups (Figure 2A). The lung cell IL-6 and IL-17 production was higher in the BCG-vaccinated non-boosted mice (451 ± 33 pg/mL) than in the BCG-vaccinated/rHBHA-boosted (271 ± 37) and in the PBS control mice (133 ± 7 pg/mL) after i.n. infection. No difference in lung cell IL-17 production between boosted and non-boosted mice was seen (Figure 2A). 

Although the lung cells from BCG-vaccinated/rHBHA-boosted mice infected by the aerosol route with *M. tuberculosis* produced a similar cytokine pattern (IL-12, IFN-*γ*, IL-6, and IL-17) as those from mice infected by i.n. route, there were no statistically significant differences between rHBHA-boosted and non-boosted animals (Figure 2B). Together these results suggest that the rHBHA boosting effect of BCG-induced protective immunity after i.n. *M. tuberculosis* infection correlates essentially with high IL-12 production (Figure 2A), since, after aerosol infection, lung cells from BCG-vaccinated/rHBHA-boosted mice produced a lower magnitude of IL-12 (Figure 2B). Thus, the absence of the methylation pattern in the C-terminal domain does not affect the induction of cytokines important for the T cell memory response but is important for the magnitude, specifically of IL-10 and IL-12.

## 4. Discussion

The natural methylation in the C-terminal domain of mycobacterial HBHA [15, 16] plays an important role in T cell antigenicity and protective immunity [17–19]. In this work, we report that the methylation pattern in the C-terminal domain of HBHA is dispensable for the booster effect of the BCG-induced protective immunity against i.n. *M. tuberculosis* infection but is important to enhance protection in lungs after aerosol challenge. 

rHBHA, a protein without the C-terminal methylation, can be easily purified from recombinant *E. coli* and is able to induce strong humoral and cellular immune responses when is administered without the adjuvant DDA+MPL by the s.c. or i.n. route to BALB/c mice [22], suggesting that the *α*-helical regions in the N-terminal domain can be target of B and T cell receptors [20, 21]. However, co-administration of rHBHA plus the DDA+MPL was unable to protect adult mice from i.v. or aerosol infection with *M. tuberculosis *[17, 18]. Heterologous prime boost regimens are an efficient way to induce long-term memory cellular immune responses leading to enhanced protection against *M. tuberculosis* [5, 9, 23]. We have recently shown that both i.n. and s.c. boosting with nHBHA in the absence of the strong Th1-adjuvant (DDA+MPL) were able to significantly enhance protection against an i.n. or aerosol *M. tuberculosis* challenge in BCG-primed, newborn mice [23]. Keeping with these observations, it was conceivable to think that, under a similar prime-boost regimen, rHBHA without methylation in the C-terminal domain and without adjuvant (DDA+MPL) could enhance BCG-induced protective immunity against *M. tuberculosis* infection. The findings of this work indicate that, while the methylation pattern in the C-terminal domain of HBHA is dispensable for the rHBHA boosting effect of the BCG-induced protective immunity against i.n. *M. tuberculosis *challenge, it is essential to induce lung protection after aerosol infection of mice with *M. tuberculosis*.

Although the most desirable effect of the vaccination with live BCG is the induction of a strong pro-Th1-type CD4^+^ T cell response through the release of IFN-*γ* [12, 13, 27], several studies have shown that enhanced protection does not necessarily correlate with the presence of IFN-*γ*-producing circulating cells. As mentioned above, heterologous prime/boost strategies are a very effective strategy to enhance protection against *M. tuberculosis *because of the induction of cytokines (IL-12, IL-17, and TGF-*β*) [25, 26] that promote the development and maintenance of long-term memory T cells [28, 29]. In agreement with this, we recently reported that enhanced BCG-mediated protective immunity against *M. tuberculosis *infection correlated precisely with these cytokines in the systemic and mucosal compartments. Interestingly, in this work we have found that BCG-vaccinated/rHBHA-boosted mice were protected against i.n. *M. tuberculosis* infection, and this enhanced protection correlated with high amount of spleen and lung IL-12 production, as well as lung TGF-*β* production. In addition, IL-10 production was detected in spleen and lungs after i.n. infection, suggesting that the lack of methylation in the C-terminal domain of HBHA could influence a more complex interplay between Th1 and regulatory type cytokines, in agreement with what has been reported elsewhere concerning the effect of IL-10 on the Th1-type cellular immune response upon vaccination [30, 31]. It is possible that, in our model, the high IL-10 production after i.n. *M. tuberculosis* infection of BCG-vaccinated/rHBHA-boosted mice promotes protection specifically at the long term, an issue that deserves future investigation. Interestingly, it has been reported recently that in early *M. tuberculosis* infection regulatory T cells could be delaying the onset of effector T cells in the lung [32].

A very moderate increase in BCG-induced spleen IFN-*γ* production (independent of the route of infection, i.n. versus aerosol) of infected mice by the s.c. rHBHA boosting was found in comparison with those reported from BCG-vaccinated nHBHA/boosted [23]. In the previous report, we proposed that the nHBHA boosting effect of BCG-induced protective immunity could be through a mechanism independent of IFN-*γ* [23]. In this study, we do not know the impact of the moderate systemic IFN-*γ* production on the control of *M. tuberculosis* infection. However, from the data it is also possible that, although the reduction of bacterial load in the spleen is significant (*P* < 0.05) upon aerosol infection (Table 1), the methylated pattern in the C-terminal domain of HBHA is required to promote the development of a more sustained cellular response in the lung compartment, as it was observed for the i.n. infected mice. However, more experiments are necessary to address this issue. 

Thus, in agreement with our previous findings, an environment of IL-12 and TGF-*β* production in long-term prime-boost regimens could promote enhanced BCG-induced protective immunity to contain *M. tuberculosis* locally and at peripheral locations, suggesting that effector and memory T cells could be involved [25, 26, 33–35]. In agreement with this, it has been reported that protection against *M. tuberculosis* induced by the prime-boost either parenterally, i.n., or through other routes did not necessarily correlate with the presence of IFN-*γ*-producing circulating T cells [36, 37].

In summary, several points arise from this study: first, the data are consistent with the fact that heterologous prime-boost protocols are a very effective way to enhance BCG-induced protective immunity and, second, the dispensability of the methylation pattern in the C-terminal domain of HBHA to enhance this protection after i.n. but not after aerosol *M. tuberculosis* infection. It is the IL-12 production and not IFN-*γ* that is very important not only to promote Th1 cellular immune responses but also for the development and maintenance of long-term memory T cells of infected mice. In addition, this report provides evidence that rHBHA may be used to improve current BCG vaccination schemes in heterologous prime/boost regimens, particularly relevant for reducing the incidence of disseminated tuberculosis.

## Figures and Tables

**Table 1:** Differential recombinant HBHA boosting effect of BCG-induced protective immunity in BALB/c newborn mice against *M. tuberculosis* H37Rv infection as measured by reduced bacterial load in spleen and lungs.

Vaccine	Lung CFUs^a^	Lung protection		Spleen CFUs^a^	Spleen protection	
		Versus			Versus	
		Control	BCG		Control	BCG
Control^b^	6.24 ± 0.25			5.04 ± 0.29		
BCG^b^	5.24 ± 0.40	1.0*		4.15 ± 0.55	0.89*	
BCG-rHBHA^b^	3.98 ± 0.11	2.26*	1.26**	3.72 ± 0.12	1.32*	0.43**
Control^c^	4.50 ± 0.71			4.50 ± 0.46		
BCG^c^	3.91 ± 0.28	0.59*		3.87 ± 0.34	0.63*	
BCG-rHBHA^c^	3.91 ± 0.47	0.59*		3.15 ± 0.71	1.35*	0.72**

^a^Data are represented as mean ± standard deviations of the log_10_-transformed bacterial (CFUs) of *M. tuberculosis* per organ; ^b^intranasal challenge; ^c^aerosol challenge. Protection is calculated with respect to control of vaccination (BCG) and with respect to PBS-immunized newborn mice (control). The data reported are *significant at *P* < 0.05 with respect to control mice and **significant at *P* < 0.05 with respect to BCG control of vaccination with no boost. One representative of a total of two independent experiments is shown.
